# Imbecile Children

**Published:** 1905-02-18

**Authors:** 


### IMBECILE CHILDREN.

Dr. Fennell deals briefly with an important subject in the January issue of the *West London Medical Journal*—viz., the forecasting of the future in the case of mentally deficient children. The subject is one that appeals but little to the attention of the general medical public, perhaps because there is a desponding echo about the word "imbecile" which damps enthusiasm. But it is an unfortunate certainty that every practitioner will be called upon to indicate the prospect in such cases, and it may be not out of place to outline the varieties of imbecility and the extent of their disabilities, with the help of Dr. Fennell's experience as to the outlook.

His classification is simple and sufficiently satisfactory, viz. :—

| A. *Congenital idiocy.* | B. *Acquired idiocy.* |
|---|---|
| 1. Simple. | 1. Traumatic. |
| 2. Micro-cephalic. | 2. Inflammatory. |
| 3. Megalo-cephalic. | 3. Epileptic. |
| 4. Mongolian. | 4. Organic. |
| 5. Cretinoid. | 5. Hypertrophic. |
| 6. Due to sense-deprivation, *e.g.*, deaf-mutism. | 6. Amaurotic family idiocy. |
| | 7. Juvenile general paralysis. |

1. The "simple" idiot is the village natural. He is a heavy oaf, but with careful education becomes competent to undertake simple duties.

2. The micro cephalic idiot, in spite of being bright and quaint, lacks capacity for attention and is a disappointing pupil.

3. Megalo-cephalic idiots sometimes improve much more than the large size of their heads would lead one to believe possible.

4. Mongols are to be identified by the slant of their palpebral fissures, their brachy-cephalic skulls, and the peculiar shortness and incurvation of their little fingers. The condition is also very commonly associated with congenital morbus cordis. Mongols improve very little and can seldom be taught anything beyond the simplest offices.

5. Cretinoid idiocy is the one bright item in the catalogue, for it only requires diagnosis in order to be curable. But the diagnosis must be made in infancy if complete success is to be achieved, and it is based upon the following physical characteristics. In a typical case the tongue protrudes, the nose is depressed, the skin harsh, the hair coarse, the hands and feet broad and spade-like. In addition there are usually pads of fat in the triangles of the neck, though these are not very obvious in infancy. Thyroid feeding, if begun early and continued throughout life, renders these patients normal in all respects.

6. Idiocy due to sense-deprivation has a hopeful outlook, with this qualification that there may be in such cases a mental deficiency latent beneath that of the senses.

In general terms the prognosis in cases of congenital idiocy is better than in the acquired forms.

Acquired idiocy :—

1. By "Traumatic idiocy" we presume Dr. Fennell means particularly that associated with the spastic paralyses attributed to injury at birth. Such cases are marked by frequent fits, and the future is gloomy.

2. The commonest variety of "inflammatory" idiocy is that following posterior basic meningitis. There is little hope of improvement in this condition.

3. Several forms of imbecility are attended by epileptic seizures. If the epilepsy is the out-standing feature, the future depends upon the frequency of the attacks, and the effect of drugs upon them. Frequent seizures in spite of treatment are very ominous.

4. By "organic" idiocy Dr. Fennell implies that depending upon cerebral tumour, whether tuberculous or other. We suspect that a condition of idiocy, in the common acceptation of the word, is seldom the result of cerebral tumour, but when it occurs it is of bad prognosis.

5. Similarly, hypertrophic idiocy, due to neuroglial overgrowth, is rare enough to be negligible for practical purposes.

6. Amaurotic family idiocy is essentially a disease of Hebrews. At the age of a few months children affected with this disease become gradually weak and lose their sight. The characteristic feature is the appearance of symmetrical white patches at the maculæ luteæ, with subsequent atrophy of the optic disc. These children usually die within two years.

7. In juvenile general paralysis all the features of the adult disease are reproduced, except the grandiose delusions ; the prospect is therefore bad.

It is important to remember that all imbeciles, and especially Mongols, are peculiarly susceptible to tuberculosis, a fact not to be overlooked in estimating the prognosis ; and from a diagnostic standpoint, that delayed muscular development, chiefly marked by weakness of the back, is common to all varieties of infant idiocy.

